# Differences in the Suitable Distribution Area between Northern and Southern China Landscape Plants

**DOI:** 10.3390/plants12142710

**Published:** 2023-07-20

**Authors:** Chen Wang, Qianqian Sheng, Runan Zhao, Zunling Zhu

**Affiliations:** 1 College of Landscape Architecture, Nanjing Forestry University, Nanjing 210037, China; wangchen1999@njfu.edu.cn (C.W.); zhao-rn@njfu.edu.cn (R.Z.); 2 Southern Modern Forestry Collaborative Innovation Center, Nanjing Forestry University, Nanjing 210037, China; 3 College of Art and Design, Nanjing Forestry University, Nanjing 210037, China

**Keywords:** landscape plants, northern and southern China, climate change, species distribution model, suitable distribution area change

## Abstract

Climate change, a global biodiversity threat, largely influences the geographical distribution patterns of species. China is abundant in woody landscape plants. However, studies on the differences in the adaptive changes of plants under climate change between northern and southern China are unavailable. Therefore, herein, the MaxEnt model was used to predict changes in the suitable distribution area (SDA) and dominant environmental variables of 29 tree species under two climate change scenarios, the shared socioeconomic pathways (SSPs) 126 and 585, based on 29 woody plant species and 20 environmental variables in northern and southern China to assess the differences in the adaptive changes of plants between the two under climate change. Temperature factors dominated the SDA distribution of both northern and southern plants. Southern plants are often dominated by one climatic factor, whereas northern plants are influenced by a combination of climatic factors. Northern plants are under greater pressure from SDA change than southern plants, and their SDA shrinkage tendency is significantly higher. However, no significant difference was observed between northern and southern plants in SDA expansion, mean SDA elevation, and latitudinal change in the SDA mass center. Future climate change will drive northern and southern plants to migrate to higher latitudes rather than to higher elevations. Therefore, future climate change has varying effects on plant SDAs within China. The climate change intensity will drive northern landscape plants to experience greater SDA-change-related pressure than southern landscape plants. Therefore, northern landscape plants must be heavily monitored and protected.

## 1. Introduction

The climate is rapidly changing in the twenty-first century [1,2], and species respond primarily through adaptation, migration, and extinction [3,4]. Climate change alters species’ suitable distribution areas (SDAs) [5]. Plants that do not adapt to the rapidly changing climate or evolve at a rate slower than that of climate change will experience SDA reduction or even extinction [6]. Climate change [7,8], anthropogenic activities [9], and variations in the sensitivity of different species to climate change will affect future species’ SDAs. Climate change is a major threat to biodiversity [10]. Currently, several studies have focused on the changes in SDAs due to climate change and variations in species sensitivity to climate change within China [11,12]. However, the differences in plant SDAs in northern and southern China under climate change remain poorly understood.

China is rich in landscape plant resources, and various plant species are widely used in modern urban greening. Green space creation in urban landscapes has positively influenced the residents’ physiological health. Green space exposure can reduce the prevalence of hypertension and respiratory diseases [13,14] and maintain people’s mental health [15,16]. A diversity in landscape plant species improves the outcomes of urban green spaces, influencing human health and well-being [17] while promoting biodiversity in an urban environment [18]. Woody landscape plants, particularly trees and shrubs, play a crucial role in landscape design. The adaptive capability of landscape plants under climate change helps domesticate garden woody flowers’ introduction, regional plant landscape configuration design, regional species diversity, and plant ecological community stability.

Current species distribution models are extensively used for endangered plant and animal conservation [19,20], species invasion [21,22], and for studying the effects of environmental change on species distribution and diversity patterns [23]. Commonly used species distribution models include Bioclim [24], Domain [25], Garp [26], and MaxEnt. The MaxEnt model was developed by Phillips et al. based on the maximum entropy principle using JAVA in 2004 [27]. MaxEnt is more versatile and effective than other species distribution modeling algorithms. 

Shared socioeconomic pathways (SSPs) can better characterize the relationship between socioeconomic development and climate scenarios [28], encompass more specific future climate cycles, and provide simulation results closer to real observations compared to CMIP5 [29].

In this study, the species distribution model was used to study the changes in plant SDAs of northern and southern China under current and future climate change. Fourteen northern and fifteen southern plants were used as samples to determine the differences in their SDAs change under climate change. Additionally, the trends of SDA size, mean SDA elevation, and latitudinal change in the SDA mass center of these species were compared for four different periods (2021–2040, 2041–2060, 2061–2080, and 2081–2100) under scenario SSP126, where there is a slow CO_2_ increase and scenario SSP585, where there is a rapid CO_2_ increase to determine the sensitivity of northern and southern landscape plants to climate change. Overall, the findings of this study will serve as a reference for future landscape plant application and conservation in northern and southern China.

## 2. Results

### 2.1. Normality Test Results

The normality test results for the SDA change data are shown in Tables S1–S8. The homogeneity test of variance for the data conforming to a normal distribution (*p* > 0.05) for both northern and southern landscape plant change data suggested that an equal variance of normally distributed SDA change data was attained (Table S9). Subsequently, the data conforming to normal distribution were subjected to differential analysis using the “independent samples *t*-test” command. However, the “double independent sample test” command was used with normal data on one side or non-normal on both sides for differential analysis.

### 2.2. Differences in the Dominant Climatic Factors between Northern and Southern Landscape Plants

**Figure 1:**
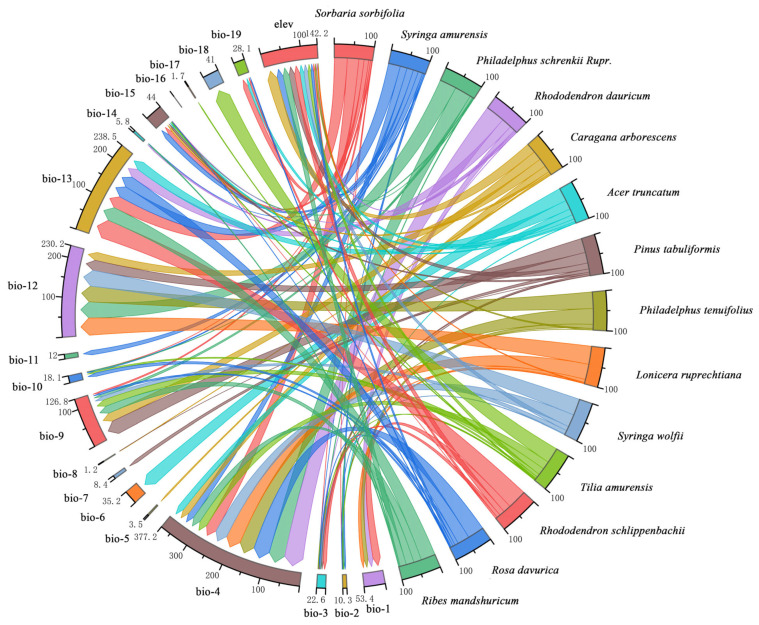
Chord diagram of the cumulative contribution of dominant climatic factors in northern landscape plants.

**Figure 2:**
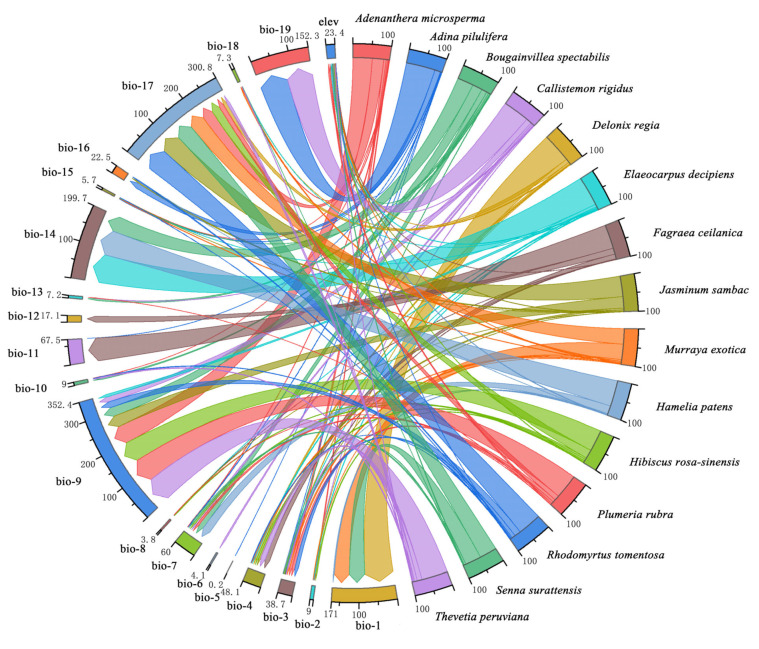
Chord diagram of the cumulative contribution of dominant climatic factors in southern landscape plants.

The specific interpretation of bioclimatic variables is shown in Table S10. The dominant climatic factors between northern and southern landscape plants are shown in the table (Tables S11 and S12). The mean highest contribution of the climatic factors in northern landscape plants was 40.66% (range: 25.90–51.50%), and SDA distribution was primarily influenced by bio-4 (temperature seasonality), bio-9 (mean temperature of the driest quarter), bio-12 (annual precipitation), bio-13 (precipitation of the wettest month), and elev (elevation), as the cumulative contribution of these influencing factors was higher in northern landscape plants (Figure 1). The mean highest contribution of the climatic factors in southern landscape plants was 64.45% (range: 43.60–82.50%), and the SDA distribution was primarily influenced by bio-9 (mean temperature of the driest quarter), bio-14 (precipitation of the driest month), bio-17 (precipitation of the driest quarter), bio-19 (precipitation of the coldest quarter), and bio-1 (annual mean temperature) (Figure 2).

### 2.3. SDA Prediction Accuracy

**Figure 3:**
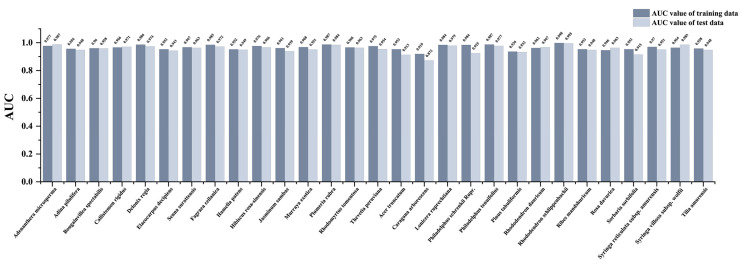
AUC values of MaxEnt model results of northern and southern landscape plants.

The AUC values generated in the model results’ file were used to validate the model predictions. In addition, the average AUC values of the models were calculated for each climatic condition. The AUC values of habitat distribution for all models were high, ranging from 0.882 to 0.998 (all above 0.8) (Figure 3). The results indicate that MaxEnt has high prediction accuracy for northern and southern landscape plant SDAs [30] and adequately simulates these SDAs under climate change scenarios.

### 2.4. Changes in SDA Expansion and Shrinkage in Northern and Southern Landscape Plants under Climate Change

**Figure 4:**
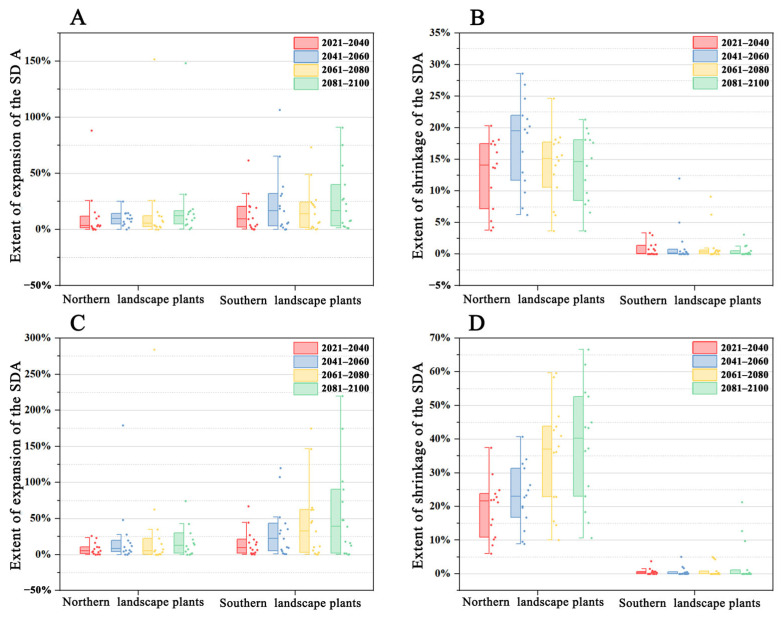
Overall SDA change trend of northern and southern landscape plants. (**A**) SDA expansion trend of northern and southern landscape plants under climate scenario 126; (**B**) SDA shrinkage trend of northern and southern landscape plants under climate scenario 126; (**C**) SDA expansion trend of northern and southern landscape plants under climate scenario 585; (**D**) SDA shrinkage trend of northern and southern landscape plants under climate scenario 585.

Except for the 2061–2080 period, the expansion trend of northern and southern landscape plants under SSP126 showed a steady increase compared with the current climate scenario (Figure 4). However, the overall shrinkage trend differed between northern and southern landscape plants. The overall SDA shrinkage trends of southern and northern landscape plants peaked in the 2041–2060 period and then gradually declined, but that of the northern landscape plant rose again in the 2081–2100 period. In addition, the overall expansion and overall shrinkage trends of the northern and southern landscape plants steadily increased under climate scenario 585. Moreover, the shrinkage expansion trend was more pronounced in both northern and southern landscape plants compared to that under climate scenario 126.

**Figure 5:**
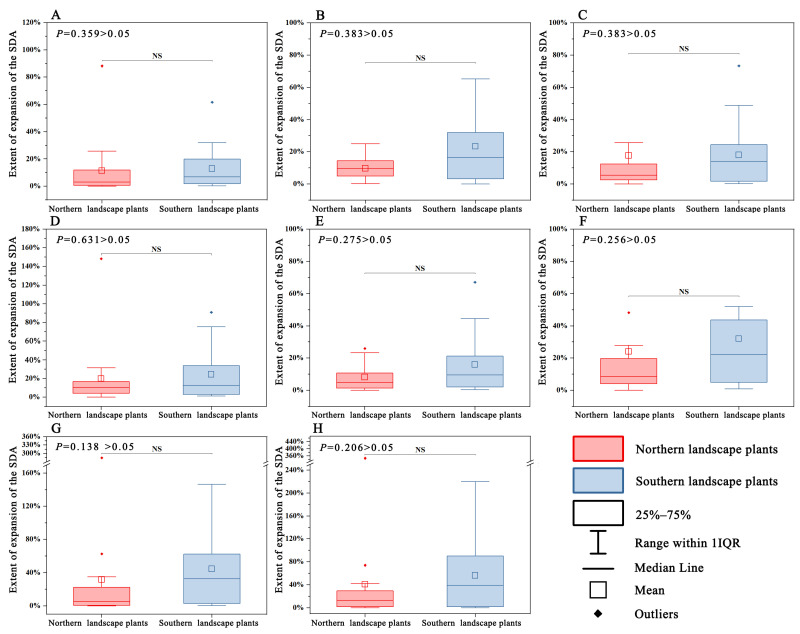
Differences in the SDA expansion trend between northern and southern landscape plants. (**A**–**D**) 2021–2040 time period under climate scenario 126 to 2081–2100 time period under climate scenario 126; (**E**–**H**) 2021–2040 period under climate scenario 585 to 2081–2100 period under climate scenario 585. (NS: Not Significant, the same below).

**Figure 6:**
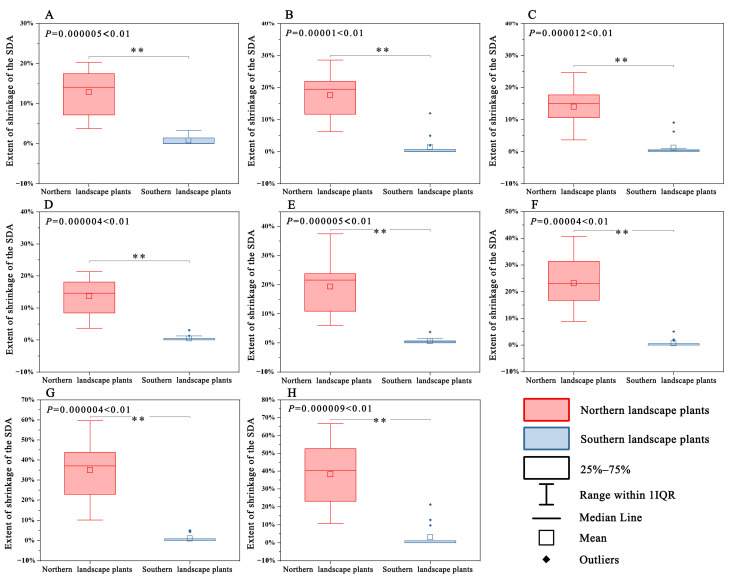
Differences in the SDA shrinkage trend between northern and southern landscape plants. (**A**–**D**) 2021–2040 period under climate scenario 126 to 2081–2100 period under climate scenario 126; (**E**–**H**) 2021–2040 period under climate scenario 585 to 2081–2100 period under climate scenario 585. (**: *p* < 0.01).

No significant difference was observed in the SDA expansion trend between northern and southern landscape plants for all four periods under climate scenario 126 and climate scenario 585 (*p* > 0.05). However, the shrinkage trend was significantly higher (*p* < 0.01) in northern landscape plants than in southern landscape plants (Figure 5 and Figure 6).

### 2.5. Changes in Mean SDA Elevation of Northern and Southern Landscape Plants under Climate Change Scenarios

**Figure 7:**
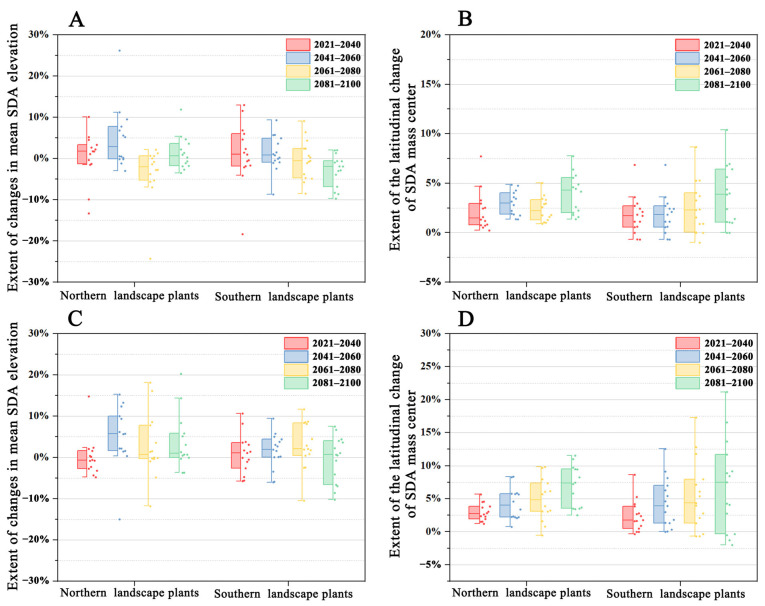
Overall SDA change trend of northern and southern landscape plants. (**A**) Mean SDA elevation change trend of northern and southern landscape plants under climate scenario 126; (**B**) latitudinal change in the SDA mass center of northern and southern landscape plants under climate scenario 126; (**C**) mean SDA elevation change trend of northern and southern landscape plants under climate scenario 585; (**D**) latitudinal change in the SDA mass center of northern and southern landscape plants under climate scenario 585.

**Figure 8:**
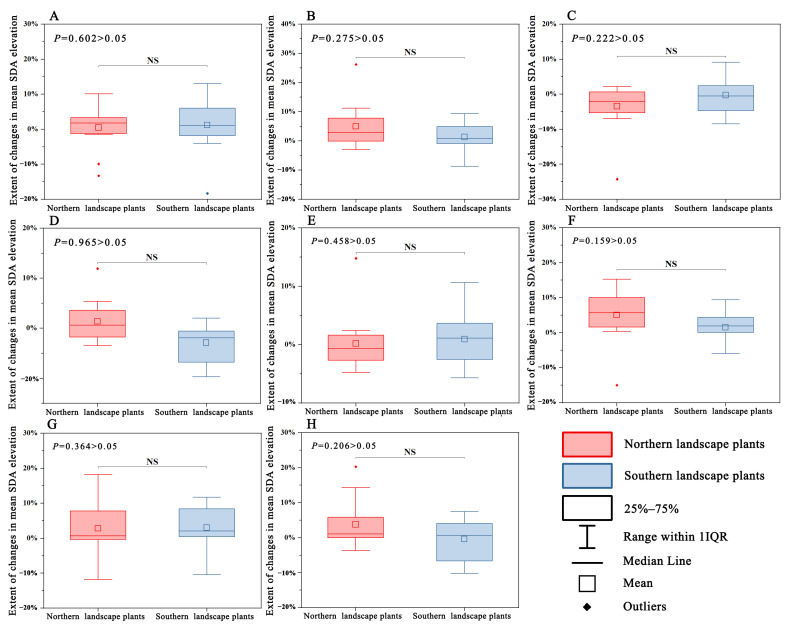
Differences in the mean SDA elevation change trend between northern and southern landscape plants. (**A**–**D**) 2021–2040 period under climate scenario 126 and 2081–2100 period under climate scenario 126; (**E**–**H**) 2021–2040 period under climate scenario 585 and 2081–2100 period under climate scenario 585.

The mean SDA elevation change trends of the northern and southern landscape plants under both climate scenarios 126 and 585 were different compared with the current climate scenario (Figure 7). Under climate scenario 126, the mean SDA elevation change magnitude in the southern landscape plants peaked in 2020–2040 and then gradually declined. In contrast, in the northern landscape habitat, it peaked in the 2040–2060 period, dropped to a minimum in the 2060–2080 period, and rose again in the 2080–2100 period. Under climate scenario 585, the mean SDA elevation change magnitude in the southern landscape peaked in the 2060–2080 period and dropped to a minimum in the 2080–2100 period. The mean SDA elevation change magnitude in the northern landscape was the same as that under climate scenario 126, exhibiting a rising–declining–rising trend; however, it was at its lowest during 2020–2040. No significant difference was observed in the elevation changes for the four periods under both climate scenarios (*p* > 0.05) (Figure 8).

### 2.6. Latitudinal Change in the SDA Mass Center of Northern and Southern Landscape Plants under Climate Change Scenarios

The overall latitudinal change in the SDA mass center of northern and southern landscape plants under climate change scenario 126 showed an increasing trend compared to the current climate scenario (Figure 7). Nevertheless, distinct variations were observed in the SDA mass center changes between the northern and southern landscape plants. For southern landscape plants, it showed a continuously increasing trend, reaching a peak during 2081–2100. However, a rising–declining–rising trend was observed in the latitudinal change in the SDA mass center of northern landscape plants, which also peaked during 2081–2100. The overall latitudinal change in the SDA mass center of the northern and southern landscape plants showed an increasing trend under climate scenario 585.

**Figure 9:**
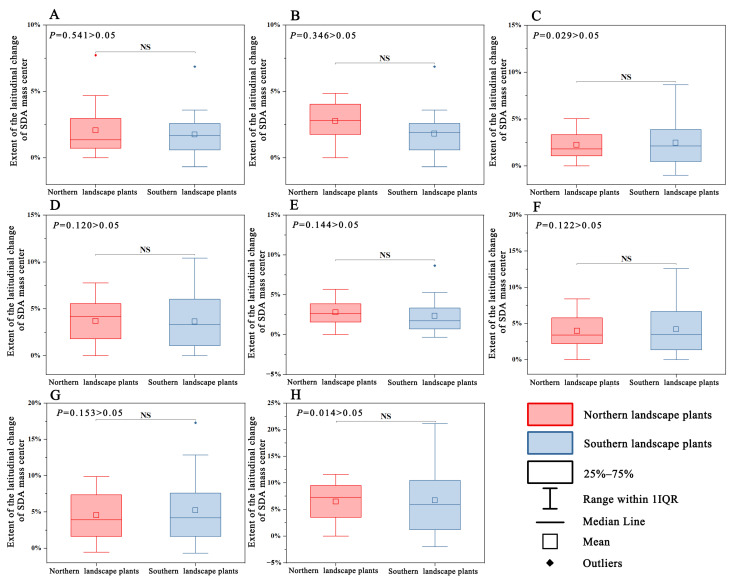
Differences in the latitudinal change in SDA mass center between northern and southern landscape plants. (**A**–**D**) 2021–2040 period under climate scenario 126 to 2081–2100 period under climate scenario 126; (**E**–**H**) 2021–2040 period under climate scenario 585 to 2081–2100 period under climate scenario 585.

No significant difference was observed in the latitudinal change in the SDA mass center for the four time periods under scenarios 126 and 585 (*p* > 0.05) (Figure 9).

## 3. Discussion

Environmental factors, such as climate change [7,8], human activities [31,32], biological factors [33], and soils [34], influence species’ geographical distribution. Climate and its variations are directly related to species distribution and biodiversity patterns [10,23,35]. In this study, a species distribution model was used to simulate the SDAs of northern and southern landscape plants under two climate change situations: the optimistic slow CO_2_ increase in the SSP126 scenario and the pessimistic rapid CO_2_ increase in the SSP585 scenario. The objective was to assess differences in overall trends in the geographical distribution of plants in different regions within the Chinese territory.

Climatic factors that dominate the SDAs of northern and southern landscape plants and their contribution rates are significantly different. The mean highest contribution rate of climatic factors for northern landscape plants was 40.66% (range: 25.90–51.50%), in which no phenomenon of one climatic factor dominating SDA distribution was observed. The same trend was observed in previous studies on 12 constructive tree species in northeastern China [36], *Ammopiptanthus mongolicus* in China [37], and mingled forests [38]. The SDA distribution of northern landscape plants was influenced by bio-4 (temperature seasonality) to the greatest extent (cumulative contribution = 377.2%), followed by bio-12 (annual precipitation) and bio-13 (precipitation of wettest month) (cumulative contribution = 238.5% and 230.2%, respectively). Northern landscape plants exhibited an overall temperature-dominated condition. 

Climatic factors with the highest contribution for southern landscape plants ranged from 43.60% to 82.50%, with a mean value of 64.45%, in which bio-9 (mean temperature of the driest quarter) almost dominated the SDA distribution. Similar trends were observed in previous studies on Chinese endemic viburnum [35], fir [39], and Sichuan pepper [40] in the Chinese range. The SDA distribution of southern landscape plants was influenced by bio-9 (cumulative contribution = 352.4%), followed by bio-17 (precipitation of the driest quarter), and bio-14 (precipitation of the driest month) (cumulative contribution = 300.8% and 199.7%, respectively). Thus, large differences were present in the composition and contribution of climatic factors between northern and southern landscape plants. Temperature factors dominated the SDAs of both northern and southern landscape plants, which was consistent with the results of studies on European forest tree species [41] and woody plants in Yunnan, China [42]. However, temperature factors differed considerably between northern and southern landscape plants, with northern landscape plants most affected by seasonal variations in temperature.

The difference in SDA changes between northern and southern landscape plants was primarily due to the SDA shrinkage trend. Climate warming poses a greater threat to northern Chinese landscape plants. In both climate scenarios, the SDA shrinkage trend was significantly lower for southern landscape plants than for northern landscape plants in all eight periods (Figure 6). The overall SDA shrinkage trend of northern and southern landscape plants was more intense under climate scenario 585 than under climate scenario 126, increasing from 0.45–1.40% and 12.86–17.62% to 0.61–3.03% and 19.32–38.19% in the four periods, respectively (Figure 6). This finding is consistent with those of previous studies [35,43]. The overall SDA shrinkage trend of southern landscape plants was more intensified compared to northern landscape plants. The SDA expansion trends of northern and southern landscape plants were not statistically significantly different (Figure 5). However, according to the general trend of SDA change between northern and southern plants (Figure 4 and Figure 7), the SDA expansion of southern landscape plants showed a better trend than that of northern landscape plants, which may be attributed to the heterogeneous climate change rate [44]. The climate change rate is more rapid in flat landscapes at high latitudes than in mountainous areas with low latitudes. The southern landscape plants selected for this study had a higher average elevation in their current climate SDA compared to northern landscape plants. Therefore, northern landscape plants experience a more rapid rate of climate change. In addition, northern landscape plants are dominated by multiple climatic factors. Therefore, any climatic factor that changes rapidly beyond the plant’s suitable distribution threshold can severely affect the SDA change in northern landscape plants. Based on the SDA expansion and shrinkage trends, northern landscape plants will experience a greater SDA change pressure under climate change scenarios compared to southern landscape plants.

Future climate change will drive species to migrate to higher latitudes [23,43]. This study revealed that the overall latitudinal change in the SDA mass center of both northern and southern landscape plants showed a northward shift. In addition, the SDA migration trend will increase with climate warming, from the mean range of 2.21–3.95% and 1.76–3.70% under climate scenario 126 to 2.99–6.92% and 2.41–6.95% under climate scenario 585 in the four periods, respectively. However, the differences between northern and southern plants were not significant. Moreover, the mean SDA elevation change trend of both northern and southern landscape plants increased and declined, which was inconsistent with the results of previous studies demonstrating that plants will migrate to higher elevations under climate warming [5,45]. However, observational studies have revealed that plants also migrate to lower elevations due to climate warming [46,47]. Furthermore, changes in the SDAs of plants along elevational gradients are not always attributed to climate change [31]. It is hypothesized that a single climate change factor could drive plant species to migrate to lower elevations. Uneven climate change rates [44], large latitudinal spans between northern and southern China, significant topographic variations, and large environmental differences in SDAs among plant species have resulted in non-uniform trends in mean SDA elevation changes. Therefore, the mechanisms of changes in the elevation gradients of plant species due to climate change within China need further refinement.

## 4. Material and Methods

### 4.1. Sample Sources

The study selected 29 woody plant species as the research subjects, and their specimen distribution points are shown in Table 1. The classification criteria for distinguishing between northern and southern plant groups were based on their extensive distribution in areas located north of the Qinling Mountains-Huaihe River region, where the average monthly temperature in January is below 0 °C.

### 4.2. Data Collection

Distribution data were obtained from the Chinese Virtual Herbarium (https://www.evh.ac.cn/, accessed on 1 June 2022) and Global Biodiversity Information Facility (https://www.gbif.org/, accessed on 1 June 2022). Data with latitude and longitude records were used directly. However, distribution points with location records were identified at the township administrative level using Gaode Map to obtain latitude and longitude data. Specimen data without images or with ambiguous information were eliminated.

Climate and elevation data were obtained from the World Climate Database (https://worldclim.org/, accessed on 1 June 2022). Climate data from WorldClim v2.1, with a 2.5’ spatial resolution, were utilized to represent both recent (1970–2000) and future (2081–2100) climatic conditions, incorporating 19 bioclimatic variables. Future climate data were selected in the BCC-CSM2-MR model for two different scenarios. The first climate change scenario was the optimistic SSP126 scenario where there are declines in CO_2_ production immediately, reaching net 0 by 2075, such that there would be additional radiative forcing of 2.6 W/m^2^ by the year 2100. The second climate change scenario was the pessimistic SSP585 scenario where CO_2_ increases even more than it has been happening recently, with CO_2_ production reaching a peak late this century, leading to a substantial additional radiative forcing of 8.5 W/m^2^ by 2100. The BCC-CSM2-MR model, the latest medium–resolution climate system model developed by the National Climate Center of China, has significantly improved simulation performance compared with the antecedent BCC-CSM-1.1 m in terms of the annual mean climate distribution of precipitation in China [48,49].

### 4.3. Pre-Processing of Specimen Point Data

**Figure 10:**
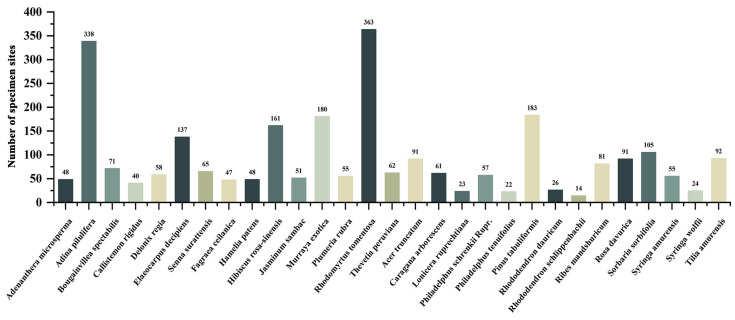
Number and distribution of specimen points.

Multiple specimen point distributions within the same raster will overfit the maximum entropy model simulation results due to the same climatic variable data within the same raster. Therefore, the collected distribution data were filtered using ENMTools [50], which automatically matches the resolution size of the climate variable layers used for the analysis and eliminates redundant specimen point data from the same raster. After redundant data elimination, 2649 final distribution point data were obtained for MaxEnt model prediction analysis (Figure 10).

### 4.4. Environment Variable Data Processing

The models constructed based on all 19 bioclimatic variables may overfit prediction results due to possible correlations among them. Therefore, correlation analysis was performed on environmental data using ENMTools, where correlation coefficients > 0.75 suggested a high degree of correlation between the two variables. Additionally, the jackknife method was used to analyze the contribution of each climatic variable to the model prediction results. The climatic variable with higher contribution was retained for SDA prediction.

### 4.5. Model Construction

The MaxEnt model (version 3.4.4) was employed to establish relationships between environmental variables and species distribution data for the selected species, aiming to determine suitable distribution areas for each species under current and future climatic conditions. In comparison to other species distribution models, the MaxEnt model yields superior simulation results by utilizing only environmental variables and a limited amount of point data. In this study, there are some plants with less distribution point data, so we chose the MaxEnt model as the modeling method. In total, 75% of the distribution data were utilized for model training, while the remaining 25% were reserved for evaluating the predictive accuracy of the model. The importance of each environmental factor in modeling the species distribution was assessed using jackknife analyses, wherein the contribution rates generated from the analyses were employed as indicators to measure the significance of bioclimatic variables.

### 4.6. SDA Change

The MaxEnt model simulation results were imported into ArcGIS software and converted to 0/1 binary maps with a threshold of 0.1 using the “quick reclassify to binary” tool in SDM Toolbox v2.4 [51], with 0 representing non-SDA and 1 representing SDA. The “distribution changes between binary SDMs” tool in SDM Toolbox was used to calculate the changes in SDA distribution caused by the climate change scenarios. The results were generated as a statistical file of the SDA range change drawings, where values “−1” represents SDA expansion, “0” represents non-SDA in both periods, “1” represents stable SDA, and “2” represents SDA shrinkage. Changes in the SDA mass center of landscape plants due to climate change scenarios were calculated using the “centroid changes (lines)” tool in SDM Toolbox. The geometric distribution in the attribute table was used to calculate the latitude and longitude data of the mass center in the WGS 1984 coordinate system. The mean SDA elevation data were extracted using the “extract by mask” command of “spatial analyst tools” in ArcGIS, using the SDA range as a mask.

The relative magnitude of change was used to assess SDA change as follows: 

SDA expansion magnitude = SDA expansion/SDA shrinkage + SDA maintenance

SDA shrinkage magnitude = SDA shrinkage/SDA shrinkage + SDA maintenance

Elevation change magnitude = average elevation of future SDA − average elevation of current SDA/average elevation of current SDA

Latitude change magnitude = latitude of future SDA mass center − latitude of current SDA mass center/latitude of future SDA mass center.

### 4.7. Statistical Analysis

SPSS software was used to analyze the SDA change data to study the differences in SDA change between northern and southern landscape plants. Normality tests were performed on the SDA change data. The Shapiro–Wilk test was used to determine the normal distribution of data because the sample numbers of northern and southern landscape plant species were 14 and 15, respectively. Finally, a homogeneity test of variance was conducted on the data that followed a normal distribution to select the most appropriate method for analyzing the differences.

## 5. Conclusions

In this study, significant differences were observed in the composition of dominant climatic factors affecting the SDAs of northern and southern landscape plants. Southern landscape plants are more likely to be primarily dominated by one climatic factor, whereas northern landscape plants are primarily affected by combinations of climatic factors. Although the SDA shrinkage trend of northern plants is highly significant compared to that of southern plants, the SDA expansion was not significantly different. According to the predicted results of future SDAs, northern landscape plants will experience greater pressure from SDA changes. In addition, both northern and southern landscape plants show a northward trend, and the SDA elevation change trend of both landscape plants presents a complex pattern. The findings of this study reveal that the SDA changes in northern and southern plants must be closely monitored, and northern plants require greater attention compared to southern plants.

## Figures and Tables

**Table 1:** Northern and southern landscape plant species and their distribution.

Species	Occurrence (Provinces)
*Sorbaria sorbifolia*	Heilongjiang, Inner Mongolia, Jilin, Liaoning, Hebei, Beijing, Shandong, Shanxi, Henan, Shaanxi, Anhui, Sichuan
*Syringa reticulata* subsp. *amurensis*	Heilongjiang, Inner Mongolia, Jilin, Liaoning, Hebei, Beijing, Shanxi, Gansu
*Philadelphus schrenkii*	Heilongjiang, Jilin, Liaoning, Hebei, Shaanxi
*Rhododendron dauricum*	Heilongjiang, Inner Mongolia, Jilin, Liaoning
*Caragana arborescens*	Heilongjiang, Inner Mongolia, Xinjiang, Jilin, Liaoning, Hebei, Beijing, Shandong, Shanxi, Shaanxi, Gansu, Qinghai
*Acer truncatum*	Liaoning, Hebei, Beijing, Shandong, Shanxi, Shaanxi, Ningxia, Gansu, Henan
*Pinus tabuliformis*	Inner Mongolia, Liaoning, Hebei, Beijing, Tianjing, Shandong, Shanxi, Shaanxi, Ningxia, Gansu, Qinghai, Henan, Hubei, Chongqing, Sichuan, Hunan, Guizhou,
*P. tenuifolius*	Heilongjiang, Jilin, Liaoning
*Lonicera ruprechtiana*	Heilongjiang, Jilin, Liaoning
*Syringa villosa* subsp. *wolfii*	Heilongjiang, Jilin, Liaoning, Beijing, Shanxi, Shaanxi
*Tilia amurensis*	Heilongjiang, Jilin Liaoning, Hebei, Beijing, Shandong, Shaanxi, Gansu, Henan
*R. schlippenbachii*	Liaoning
*Rosa davurica*	Heilongjiang, Inner Mongolia, Jilin, Liaoning, Hebei, Beijing, Shanxi
*Ribes mandshuricum*	Heilongjiang, Jilin, Liaoning, Hebei, Beijing, Shandong, Shanxi, Shaanxi, Gansu, Henan
*Adenanthera microsperma*	Jiangxi, Guizhou, Taiwan, Fujian, Guangdong, Guangxi, Yunan, Hainan
*Adina pilulifera*	Anhui, Zhejiang, Jiangxi, Hunan, Guizhou, Fujian, Guangdong, Hong Kong, Guangxi, Hainan
*Bougainvillea spectabilis*	Chongqing, Sichuan, Taiwan, Fujian, Jiangxi, Guizhou, Guangdong, Guangxi, Yunnan, Hainan
*Callistemon rigidus*	Shanghai, Zhejiang, Fujian, Guizhou, Guangdong, Hong Kong, Guangxi, Yunnan, Hainan
*Delonix regia*	Chongqing, Sichuan, Fujian, Guangdong, Hong Kong, Guangxi, Yunnan, Hainan
*Elaeocarpus decipiens*	Shanghai, Zhejiang, Hubei, Chongqing, Taiwan, Fujian, Jiangxi, Hunan, Guizhou, Guangdong, Hong Kong, Guangxi, Yunnan, Hainan
*Fagraea ceilanica*	Fujian, Guangdong, Hong Kong, Guangxi, Yunnan, Hainan
*Jasminum sambac*	Zhejiang, Hubei, Sichuan, Taiwan, Fujian, Hunan, Guizhou, Guangdong, Hong Kong, Guangxi, Yunnan, Hainan
*Murraya exotica*	Taiwan, Fujian, Hunan, Guizhou, Guangdong, Hong Kong, Guangxi, Yunnan, Hainan
*Hamelia patens*	Zhejiang, Fujian, Hunan, Guizhou, Guangdong, Hong Kong, Guangxi, Yunnan, Hainan
*Hibiscus rosa-sinensis*	Fujian, Hunan, Guizhou, Guangdong, Hong Kong, Macao, Guangxi, Yunnan, Hainan
*Plumeria rubra*	Taiwan, Fujian, Guangdong, Hong Kong, Guangxi, Yunnan, Hainan
*Rhodomyrtus tomentosa*	Zhejiang, Taiwan, Fujian, Jiangxi, Hunan, Guizhou, Guangdong, Hong Kong, Macao, Guangxi, Yunnan, Hainan
*Senna surattensis*	Chongqing, Taiwan, Fujian, Jiangxi, Guizhou, Sichuan, Guangdong, Hong Kong, Guangxi, Yunnan, Hainan
*Thevetia peruviana*	Chongqing, Sichuan, Taiwan, Fujian, Jiangxi, Guizhou, Guangdong, Hong Kong, Guangxi, Yunnan, Hainan

## Data Availability

All databases (sources and references) are described in the Section 4.

## Supplementary Materials

The following supporting information can be downloaded at: https://www.mdpi.com/article/10.3390/plants12142710/s1, Table S1: Normal test for SDA expansion data of northern landscape plants; Table S2: Normal test for SDA expansion data of southern landscape plants; Table S3: Normal test for SDA shrinkage data of northern landscape plants; Table S4: Normal test for SDA shrinkage data of southern landscape plants; Table S5: Normal test for mean SDA elevation change data of northern landscape plants; Table S6: Normal test for mean SDA elevation change data of southern landscape plants; Table S7: Normal test for latitudinal change in the SDA mass center data of northern landscape plants; Table S8: Normal test for latitudinal change in the SDA mass center data of southern landscape plants; Table S9: Homogeneity test of variance; Table S10: Interpretation of bioclimatic variables; Table S11: Dominant climatic factors in northern landscape plants; Table S12: Dominant climatic factors in southern landscape plants.

## References

[1] Diffenbaugh N.S., Field C.B. (2013). Changes in Ecologically Critical Terrestrial Climate Conditions. Science.

[2] Moritz C., Agudo R. (2013). The future of species under climate change: Resilience or decline?. Science.

[3] Aitken S.N., Yeaman S., Holliday J.A., Wang T., Curtis-McLane S. (2008). Adaptation, migration or extirpation: Climate change outcomes for tree populations. Evol. Appl..

[4] Jump A.S., Penuelas J. (2005). Running to stand still: Adaptation and the response of plants to rapid climate change. Ecol. Lett..

[5] Kelly A.E., Goulden M.L. (2008). Rapid shifts in plant distribution with recent climate change. Proc. Natl. Acad. Sci. USA.

[6] Aitken S.N., Bemmels J.B. (2016). Time to get moving: Assisted gene flow of forest trees. Evol. Appl..

[7] Bellard C., Bertelsmeier C., Leadley P., Thuiller W., Courchamp F. (2012). Impacts of climate change on the future of biodiversity. Ecol. Lett..

[8] Chen I.-C., Hill J.K., Ohlemüller R., Roy D.B., Thomas C.D. (2011). Rapid range shifts of species associated with high levels of climate warming. Science.

[9] Tuholske C., Tane Z., López-Carr D., Roberts D., Cassels S. (2017). Thirty years of land use/cover change in the Caribbean: Assessing the relationship between urbanization and mangrove loss in Roatán, Honduras. Appl. Geogr..

[10] Dawson T.P., Jackson S.T., House J.I., Prentice I.C., Mace G.M. (2011). Beyond Predictions: Biodiversity Conservation in a Changing Climate. Science.

[11] Cao B., Bai C., Zhang M., Lu Y., Gao P., Yang J., Xue Y., Li G. (2022). Future landscape of renewable fuel resources: Current and future conservation and utilization of main biofuel crops in China. Sci. Total Environ..

[12] Zhao X., Lei M., Wei C., Guo X. (2022). Assessing the suitable regions and the key factors for three Cd-accumulating plants (*Sedum alfredii*, *Phytolacca americana*, and *Hylotelephium spectabile*) in China using MaxEnt model. Sci. Total Environ..

[13] Donovan G.H., Butry D.T., Michael Y.L., Prestemon J.P., Liebhold A.M., Gatziolis D., Mao M.Y. (2013). The relationship between trees and human health: Evidence from the spread of the emerald ash borer. Am. J. Prev. Med..

[14] Shanahan D.F., Bush R., Gaston K.J., Lin B.B., Dean J., Barber E., Fuller R.A. (2016). Health Benefits from Nature Experiences Depend on Dose. Sci. Rep..

[15] De Vries S., Verheij R.A., Groenewegen P.P., Spreeuwenberg P. (2003). Natural environments—Healthy environments? An exploratory analysis of the relationship between greenspace and health. Environ. Plan A.

[16] Thompson C.W., Roe J., Aspinall P., Mitchell R., Clow A., Miller D. (2012). More green space is linked to less stress in deprived communities: Evidence from salivary cortisol patterns. Landsc Urban Plan..

[17] Southon G.E., Jorgensen A., Dunnett N., Hoyle H., Evans K.L. (2018). Perceived species-richness in urban green spaces: Cues, accuracy and well-being impacts. Landsc. Urban Plan..

[18] Paker Y., Yom-Tov Y., Alon-Mozes T., Barnea A. (2014). The effect of plant richness and urban garden structure on bird species richness, diversity and community structure. Landsc Urban Plan..

[19] Ouyang X., Bai S., Strachan G.B., Chen A. (2022). Simulation of the potential distribution of rare and endangered Satyrium species in China under climate change. Ecol. Evol..

[20] Ye P., Zhang G., Zhao X., Chen H., Si Q., Wu J. (2021). Potential geographical distribution and environmental explanations of rare and endangered plant species through combined modeling: A case study of Northwest Yunnan, China. Ecol. Evol..

[21] Anibaba Q.A., Dyderski M.K., Jagodzinski A.M. (2022). Predicted range shifts of invasive giant hogweed (*Heracleum mantegazzianum*) in Europe. Sci. Total Environ..

[22] Wan J.Z., Wang C.J., Tan J.F., Yu F.H. (2017). Climatic niche divergence and habitat suitability of eight alien invasive weeds in China under climate change. Ecol. Evol..

[23] Sun S., Zhang Y., Huang D., Wang H., Cao Q., Fan P., Yang N., Zheng P., Wang R. (2020). The effect of climate change on the richness distribution pattern of oaks (*Quercus* L.) in China. Sci. Total Environ..

[24] Busby J.R. (1991). BIOCLIM: A bioclimate analysis and prediction system. Plant Prot. Q..

[25] Carpenter G., Gillison A.N., Winter J. (1993). DOMAIN: A flexible modelling procedure for mapping potential distributions of plants and animals. Biodivers. Conserv..

[26] Stockwell D. (1999). The GARP modelling system: Problems and solutions to automated spatial prediction. J. Geogr. Sci..

[27] Phillips S.J., Anderson R.P., Schapire R.E. (2006). Maximum entropy modeling of species geographic distributions. Ecol. Model..

[28] Moss R.H., Edmonds J.A., Hibbard K.A., Manning M.R., Rose S.K., van Vuuren D.P., Carter T.R., Emori S., Kainuma M., Kram T. (2010). The next generation of scenarios for climate change research and assessment. Nature.

[29] Taylor K.E., Stouffer R.J., Meehl G.A. (2011). An overview of CMIP5 and the experiment design. Bull. Am. Meteorol. Soc..

[30] Swets J.A. (1988). Measuring the Accuracy of Diagnostic Systems. Science.

[31] Gehrig-Fasel J., Guisan A., Zimmermann N.E. (2007). Tree line shifts in the Swiss Alps: Climate change or land abandonment?. J. Veg. Sci..

[32] Treml V., Šenfeldr M., Chuman T., Ponocná T., Demková K., Collins B. (2016). Twentieth century treeline ecotone advance in the Sudetes Mountains (Central Europe) was induced by agricultural land abandonment rather than climate change. J. Veg. Sci..

[33] Tylianakis J.M., Didham R.K., Bascompte J., Wardle D.A. (2008). Global change and species interactions in terrestrial ecosystems. Ecol. Lett..

[34] Dubuis A., Giovanettina S., Pellissier L., Pottier J., Vittoz P., Guisan A., Rocchini D. (2013). Improving the prediction of plant species distribution and community composition by adding edaphic to topo-climatic variables. J. Veg. Sci..

[35] Ying J. (2021). Predicting the Impacts of Climate Change on the Potential Distribution of Endemic Viburnum in China by MaxEnt Model. Master’s Thesis.

[36] Du Q., Wei C., Liang C., Yu J., Wang H., Wang W. (2022). Future climatic adaption of 12 dominant tree species in Northeast China under 3 climatic scenarios by using MaxEnt modeling. Sheng Tai Xue Bao.

[37] Duan Y., Wang C., Wang H., Du Z., He Y., Chai G. (2020). Predicting the potential distribution of Ammopiptanthus species in China underdifferent climates using ecological niche models. Sheng Tai Xue Bao.

[38] Wen G., Ye X., Lai W., Shi C., Huang Q., Ye L., Zhang G. (2021). Dynamic analysis of mixed forest species under climate change scenarios. Ecol. Indic..

[39] Zhao Y., Deng X., Xiang W., Chen L., Ouyang S. (2021). Predicting potential suitable habitats of Chinese fir under current and future climatic scenarios based on Maxent model. Ecol. Inform..

[40] Xu D., Zhuo Z., Wang R., Ye M., Pu B. (2019). Modeling the distribution of Zanthoxylum armatum in China with MaxEnt modeling. Glob. Ecol. Conserv..

[41] Dyderski M.K., Paz S., Frelich L.E., Jagodzinski A.M. (2018). How much does climate change threaten European forest tree species distributions?. Glob. Chang. Biol..

[42] Zhang M.-G., Zhou Z.-K., Chen W.-Y., Cannon C.H., Raes N., Slik J.W.F. (2014). Major declines of woody plant species ranges under climate change in Yunnan, China. Divers. Distrib..

[43] Guo K. (2021). Analysis on the Distribution Characteristics of Quercus Section Cyclobalanopsis. Master’s Thesis.

[44] Loarie S.R., Duffy P.B., Hamilton H., Asner G.P., Field C.B., Ackerly D.D. (2009). The velocity of climate change. Nature.

[45] Lenoir J., Gégout J.C., Marquet P.A., de Ruffray P., Brisse H. (2008). A Significant Upward Shift in Plant Species Optimum Elevation During the 20th Century. Science.

[46] Crimmins S.M., Dobrowski S.Z., Greenberg J.A., Abatzoglou J.T., Mynsberge A.R.J.S. (2011). Changes in climatic water balance drive downhill shifts in plant species’ optimum elevations. Science.

[47] Rabasa S.G., Granda E., Benavides R., Kunstler G., Espelta J.M., Ogaya R., Penuelas J., Scherer-Lorenzen M., Gil W., Grodzki W. (2013). Disparity in elevational shifts of European trees in response to recent climate warming. Glob. Chang. Biol..

[48] Wu T., Lu Y., Fang Y., Xin X., Li L., Li W., Jie W., Zhang J., Liu Y., Zhang L. (2019). The Beijing Climate Center Climate System Model (BCC-CSM): The main progress from CMIP5 to CMIP6. Geosci. Model Dev..

[49] Xin X.-G., Wu T.-W., Zhang J., Zhang F., Li W.-P., Zhang Y.-W., Lu Y.-X., Fang Y.-J., Jie W.-H., Zhang L. (2019). Introduction of BCC models and its participation in CMIP6. Adv. Clim. Chang. Res..

[50] Warren D.L., Glor R.E., Turelli M. (2010). ENMTools: A toolbox for comparative studies of environmental niche models. Ecography.

[51] Brown J.L., Anderson B. (2014). SDMtoolbox: A python-based GIS toolkit for landscape genetic, biogeographic and species distribution model analyses. Methods Ecol. Evol..

