# Supplementary material for: Differences in the Suitable Distribution Area between Northern and Southern China Landscape Plants
Source: Plants (Basel). 2023 Jul 20;12(14):2710. doi: 10.3390/plants12142710 (PMC10385631; doi:10.3390/plants12142710)
Supplement: Supplementary file 1 [file plants-12-02710-s001.zip › plants-2445718-supplementary.pdf]

Supplementary Materials

Table S1. Normal test for SDA expansion data of northern landscape plants.

Northern landscape plants	Statistics	Degree of freedom	Significance
126:2021–2040	0.544	14	0.000
126:2041–2060	0.948	14	0.536
126:2061–2080	0.449	14	0.000
126:2081–2100	0.490	14	0.000
585:2021–2040	0.853	14	0.025
585:2041–2060	0.522	14	0.000
585:2061–2080	0.468	14	0.000
585:2081–2100	0.475	14	0.000

Table S2. Normal test for SDA expansion data of southern landscape plants.

Southern landscape plants	Statistics	Degree of freedom	Significance
126:2021–2040	0.780	15	0.002
126:2041–2060	0.770	15	0.002
126:2061–2080	0.811	15	0.005
126:2081–2100	0.825	15	0.008
585:2021–2040	0.794	15	0.003
585:2041–2060	0.787	15	0.003
585:2061–2080	0.792	15	0.003
585:2081–2100	0.815	15	0.006

Table S3. Normal test for SDA shrinkage data of northern landscape plants.

Northern landscape plants	Statistics	Degree of freedom	Significance
126:2021–2040	0.894	14	0.093
126:2041–2060	0.956	14	0.654
126:2061–2080	0.953	14	0.609
126:2081–2100	0.935	14	0.353
585:2021–2040	0.959	14	0.710
585:2041–2060	0.973	14	0.912
585:2061–2080	0.945	14	0.489
585:2081–2100	0.962	14	0.764

Table S4. Normal test for SDA shrinkage data of southern landscape plants.

Southern landscape plants	Statistics	Degree of freedom	Significance
126:2021–2040	0.735	15	0.001
126:2041–2060	0.509	15	0.000
126:2061–2080	0.522	15	0.000
126:2081–2100	0.606	15	0.000
585:2021–2040	0.651	15	0.000
585:2041–2060	0.578	15	0.000
585:2061–2080	0.575	15	0.000
585:2081–2100	0.557	15	0.000

Table S5. Normal test for mean SDA elevation change data of northern landscape plants.

Northern landscape plants	Statistics	Degree of freedom	Significance
126:2021–2040	0.883	14	0.063
126:2041–2060	0.817	14	0.008
126:2061–2080	0.698	14	0.000
126:2081–2100	0.897	14	0.101
585:2021–2040	0.756	14	0.001
585:2041–2060	0.887	14	0.074
585:2061–2080	0.923	14	0.241
585:2081–2100	0.859	14	0.030

Table S6. Normal test for mean SDA elevation change data of southern landscape plants.

Southern landscape plants	Statistics	Degree of freedom	Significance
126:2021–2040	0.906	15	0.118
126:2041–2060	0.949	15	0.510
126:2061–2080	0.982	15	0.982
126:2081–2100	0.918	15	0.182
585:2021–2040	0.955	15	0.606
585:2041–2060	0.955	15	0.611
585:2061–2080	0.939	15	0.371
585:2081–2100	0.936	15	0.337

Table S7. Normal test for latitudinal change in the SDA mass center data of northern landscape plants.

Northern landscape plants	Statistics	Degree of freedom	Significance
126:2021–2040	0.825	14	0.01
126:2041–2060	0.925	14	0.257
126:2061–2080	0.929	14	0.298
126:2081–2100	0.932	14	0.322
585:2021–2040	0.952	14	0.591
585:2041–2060	0.906	14	0.139
585:2061–2080	0.964	14	0.792
585:2081–2100	0.907	14	0.144

Table S8. Normal test for latitudinal change in the SDA mass center data of southern landscape plants.

Southern landscape plants	Statistics	Degree of freedom	Significance
126:2021–2040	0.91	15	0.136
126:2041–2060	0.913	15	0.152
126:2061–2080	0.936	15	0.334
126:2081–2100	0.925	15	0.227
585:2021–2040	0.897	15	0.085
585:2041–2060	0.932	15	0.296
585:2061–2080	0.924	15	0.222
585:2081–2100	0.95	15	0.528

Table S9. Homogeneity test of variance.

Mean SDA elevation change data	Levene Statistics	Significance	Latitudinal change in the SDA mass center data	Levene Statistics	Significance
126:2021–2040	0.278	0.602	126:2041–2060	0.919	0.346
126:2081–2100	0.002	0.965	126:2061–2080	5.309	0.029
585:2041–2060	2.095	0.159	126:2081–2100	2.578	0.12
585:2061–2080	0.853	0.364	585:2021–2040	2.266	0.144
			585:2041–2060	2.543	0.122
			585:2061–2080	2.157	0.153
			585:2081–2100	6.959	0.014

Table S10. Interpretation of bioclimatic variables.

Bioclimatic variables	Interpretation
bio-1	Annual Mean Temperature
bio-2	Mean Diurnal Range (Mean of monthly (max temp - min temp))
bio-3	Isothermality (bio-2/bio-7) (×100)
bio-4	Temperature Seasonality (standard deviation ×100)
bio-5	Max Temperature of Warmest Month
bio-6	Min Temperature of Coldest Month
bio-7	Temperature Annual Range (bio-5～bio-6)
bio-8	Mean Temperature of Wettest Quarter
bio-9	Mean Temperature of Driest Quarter
bio-10	Mean Temperature of Warmest Quarter
bio-11	Mean Temperature of Coldest Quarter
bio-12	Annual Precipitation
bio-13	Precipitation of Wettest Month
bio-14	Precipitation of Driest Month
bio-15	Precipitation Seasonality (coefficient of Variation)
bio-16	Precipitation of Wettest Quarter
bio-17	Precipitation of Driest Quarter
bio-18	Precipitation of Warmest Quarter
bio-19	Precipitation of Coldest Quarter

Table S11. Dominant climatic factors in northern landscape plants.

Species	Climatic factors and contribution rate/%								
Sorbaria sorbifolia	bio-13	bio-4	bio-19	bio-15	elev	bio-9	bio-10	bio-3	bio-2
	34.1	30.2	13.9	11.9	4.9	4	0.4	0.4	0.3
Syringa reticulata subsp. *amurensis*	bio-13	bio-4	elev	bio-15	bio-11	bio-2	bio-10	bio-3	bio-14
	31.7	18.1	17.5	13.2	12	4	2.9	0.4	0.2
Philadelphus schrenkii Rupr.	bio-12	bio-4	bio-9	bio-15	bio-3	bio-10	elev		
	44.4	43.8	6.9	1.8	1.7	1.1	0.4		
Rhododendron dauricum	bio-4	bio-13	bio-1	elev	bio-14	bio-3	bio-15		
	50.9	26.8	14.1	3.6	3.6	0.5	0.4		
Caragana arborescens	elev	bio-9	bio-12	bio-4	bio-15	bio-3	bio-8		
	25.9	24.3	22.3	17.7	5.3	3.5	1.1		
Acer truncatum	bio-6	bio-13	bio-4	elev	bio-19	bio-15	bio-2		
	35.2	24.5	16.5	11.6	7.3	3.7	1.2		
Pinus tabuliformis	bio-9	bio-12	elev	bio-7	bio-15	bio-3	bio-2	bio-8	
	37.6	28.4	15.7	8.4	4.6	4.4	0.8	0.1	
Philadelphus tenuifolius	bio-12	bio-4	bio-1	elev	bio-3				
	43.8	41.1	10.9	3.9	0.3				
Lonicera ruprechtiana	bio-12	bio-4	bio-1	elev	bio-3				

	47.7	40.6	8.1	2	1.6				
Syringa villosa subsp. *wolfii*	bio-12	bio-4	elev	bio-9	bio-3				
	43.6	32.9	11.5	10.3	1.7				
Tilia amurensis	bio-18	bio-4	bio-9	elev	bio-10	bio-15	bio-17	bio-2	bio-3
	41	21.3	18.2	8.6	4.4	2.2	1.7	1.4	1.2
Rhododendron schlippenbachii	bio-13	bio-1	elev	bio-3	bio-19	bio-15			
	51.5	20.3	14.2	9.1	4.9	0.1			
Rosa davurica	bio-4	bio-13	bio-10	elev	bio-9	bio-19	bio-3	bio-15	bio-2
	43.6	31.7	9.3	6	5	2	1.3	0.8	0.3
Ribes mandshuricum	bio-13	bio-4	bio-9	elev	bio-2	bio-14			
	38.2	20.5	20.5	16.4	2.3	2			

Table S12. Dominant climatic factors in southern landscape plants.

Species	Climatic factors and contribution rate/%								
Adenanthera microsperma	bio-9	bio-17	bio-3	elev	bio-2	bio-18	bio-7	bio-15	bio-10
	52.9	31.3	6.5	3.6	2.6	1.4	1	0.4	0.4
Adina pilulifera	bio-19	bio-16	bio-15	bio-7	Elev	bio-11	bio-3	bio-5	
	79.7	15.6	1.6	1.4	0.8	0.5	0.3	0.2	
Bougainvillea spectabilis	bio-14	bio-9	bio-7	bio-13	bio-10	elev	bio-3	bio-15	
	44.6	27.3	15	5.8	3.8	1.3	1.2	0.9	
Callistemon rigidus	bio-19	bio-9	bio-7	bio-8	Elev	bio-13	bio-2		
	72.6	15.3	4.9	4.1	1.3	1	0.9		
Delonix regia	bio-1	bio-17	bio-4	bio-3	bio-18	elev	bio-2		
	81.2	10.8	4.7	1.5	0.8	0.7	0.4		
Elaeocarpus decipiens	bio-14	bio-9	bio-4	elev	bio-2	bio-3	bio-15	bio-8	bio-18
	82.5	7.2	2.9	2.6	1.8	1	0.7	0.7	0.6
Fagraea ceilanica	bio-11	bio-12	bio-4	bio-3	bio-8	elev			
	67	17.1	13.4	1.6	0.5	0.4			
Jasminum sambac	bio-17	bio-9	bio-4	bio-16	Elev	bio-3	bio-15	bio-8	
	57.3	32.1	5.1	3.7	1.2	0.4	0.1	0.1	
Murraya exotica	bio-17	bio-1	bio-3	bio-18	bio-4	bio-15	elev	bio-2	
	43.6	43.1	4.6	3.4	2.6	1.3	0.8	0.6	
Hamelia patens	bio-14	bio-7	bio-1	elev	bio-3	bio-16			
	72.6	22.5	2.6	1.9	0.3	0.1			
Hibiscus rosa-sinensis	bio-9	bio-17	bio-3	bio-7	bio-2	bio-8	elev		

	59.8	24.2	4.9	4.8	2.9	2	1.3	
Plumeria rubra	bio-9	bio-17	bio-3	bio-7	Elev	bio-8	bio-13	
	68.1	13.4	7.8	5.6	4.2	0.5	0.4	
Rhodomyrtus tomentosa	bio-17	bio-9	bio-3	bio-16	bio-10	bio-4	bio-15	elev
	65.2	17.6	9.6	3.1	2	1.4	0.7	0.4
Senna surattensis	bio-17	bio-1	bio-7	elev	bio-3	bio-18		
	47.5	44.1	4.8	1.8	1.6	0.1		
Thevetia peruviana	bio-9	bio-4	bio-17	bio-10	bio-3	elev	bio-18	
	72.1	13.1	7.5	2.8	2.5	1.1	1	
